# 5HT_2A_ and 5HT_2B_ Receptors Contribute to Serotonin-Induced Vascular Dysfunction in Diabetes

**DOI:** 10.1155/2012/398406

**Published:** 2012-12-30

**Authors:** Peter M. Nelson, Jeremy S. Harrod, Kathryn G. Lamping

**Affiliations:** ^1^Iowa City Veterans Affairs Health Care System, Research Service (151), 601 Highway 6 West, Iowa City, IA 52246, USA; ^2^Departments of Internal Medicine and Pharmacology, Roy J. and Lucille A. Carver College of Medicine, University of Iowa, Iowa City, IA 52242, USA

## Abstract

Although 5HT_2A_ receptors mediate contractions of normal arteries to serotonin (5HT), in some cardiovascular diseases, other receptor subtypes contribute to the marked increase in serotonin contractions. We hypothesized that enhanced contractions of arteries from diabetics to 5HT are mediated by an increased contribution from multiple 5HT receptor subtypes. We compared responses to selective 5HT receptor agonists and expression of 5HT receptor isoforms (5HT_1B_, 5HT_2A_, and 5HT_2B_) in aorta from nondiabetic (ND) compared to type 2 diabetic mice (DB, BKS.Cg-*Dock7* ^*m*^+/+*Lepr^db^*/J). 5HT, 5HT_2A_ (TCB2 and BRL54443), and 5HT_2B_ (norfenfluramine and BW723C86) receptor agonists produced concentration-dependent contractions of ND arteries that were markedly increased in DB arteries. Neither ND nor DB arteries contracted to a 5HT_1B_ receptor agonist. MDL11939, a 5HT_2A_ receptor antagonist, and LY272015, a 5HT_2B_ receptor antagonist, reduced contractions of arteries from DB to 5HT more than ND. Expression of 5HT_1B_, 5HT_2A_, and 5HT_2B_ receptor subtypes was similar in ND and DB. Inhibition of rho kinase decreased contractions to 5HT and 5HT_2A_ and 5HT_2B_ receptor agonists in ND and DB. We conclude that in contrast to other cardiovascular diseases, enhanced contraction of arteries from diabetics to 5HT is not due to a change in expression of multiple 5HT receptor subtypes.

## 1. Introduction

Vascular responses to serotonin (5HT), a neurohumoral factor released from activated platelets implicated in vasospasm of large coronary and cerebral arteries, are determined by multiple factors including binding to distinct receptor subtypes, species, sex, and vessel size [1–5]. The net response to serotonin in most vascular beds is a balance between effects on endothelium and vascular smooth muscle. Serotonin stimulates release of vasodilator substances such as nitric oxide by activating 5HT_1_ receptors on endothelial cells. In most arteries, serotonin-induced contractions are mediated by 5HT_2A_ receptors on vascular smooth muscle, which activates the rho/rho kinase pathway to regulate phosphorylation of myosin light chain phosphatase and myosin light chain [5, 6]. Although 5HT_1B_, 5HT_2A_, and 5HT_2B_ receptor subtypes are expressed in vasculature from normal mice [7, 8], only the contribution of 5HT_2A_ receptors has been addressed [4, 5].

Determining the specific receptor subtypes that mediate vascular responses may be key to deciphering mechanisms contributing to augmented contractile responses to serotonin observed in a variety of cardiovascular diseases. Although activation of 5HT_2A_ receptors predominates in normal arteries [4, 5], expression and function of other serotonergic receptor subtypes (primarily 5HT_1B_ and 5HT_2B_) are upregulated in hypertension and atherosclerosis contributing to greater contractions to serotonin [7–13]. We and others have previously demonstrated that arterial contractions to serotonin are markedly increased in models of type 1 and 2 diabetes [14–17]. Although the increased contraction to serotonin may be mediated, in part, by reductions in nitric oxide bioavailability, this mechanism alone cannot account for the marked hypercontractile response observed in many models of cardiovascular disease [18]. RhoA/rho kinase-mediated contractions to serotonin are increased in arteries from diabetics, but the augmented contractions are not due to a change in expression of either rhoA or rho kinase, which regulate calcium sensitivity of contractile proteins [16, 17]. One alternative hypothesis that may account for the marked increase in serotonin-induced contractions is that expression of specific serotonin receptor subtypes upregulates in diabetics to increase the magnitude of the contraction. Although one report found that 5HT_2A_ receptor expression was increased in mesenteric arteries of diabetic Otsuka Long-Evans Tokushima fatty rats [19], the role of other serotonin receptor subtypes in mediating the vascular dysfunction in diabetics has never been examined. To address this hypothesis, we compared expression levels of multiple serotonin receptors in relationship to vascular responses of nondiabetics and diabetics to selective 5HT receptor agonists and antagonists.

## 2. Methods 

### 2.1. Experimental Model

The animal protocol was approved by the Iowa City Veterans Affairs Health Care System which complied with the Guide to the Care and Use of Laboratory Animals. Male non-diabetic C57BLKS/J (ND, the genetic background for BKS.Cg-*m*+/+*Lepr* ^*db*^/J) and age-matched homozygous leptin receptor deficient diabetic (DB, BKS.Cg-*m*+/+*Lepr* ^*db*^/J) mice were obtained from Jackson Laboratories. On the day of study, weight and levels of nonfasting blood glucose were measured following euthanasia (sodium pentobarbital, 150 mg/kg ip). The weights and blood glucose levels of the DB mice were greater than ND mice (Table 1).

### 2.2. Measurement of Vascular Reactivity

Studies were performed in aorta since this allowed us to compare vascular reactivity with protein expression in the same vessel. Responses of aorta were measured using previously published methods [2]. Aorta was removed, placed in ice-cold Krebs buffer (mmol/L: NaCl 118.3, KCl 4.7, CaCl_2_ 2.5, MgSO_4_ 1.2, KH_2_PO_4_ 1.2, NaHCO_3_ 25, and glucose 5), cut into rings (3-4** **mm in length), and mounted on wires connected to a force transducer in an organ bath filled with Krebs (37°C aerated with 20% O_2_, 5% CO_2_, and balance N_2_). Tension was incrementally increased to 0.75** **g over 45–60 minutes. Contractions to serotonin (0.01 to 10 *μ*M); KCl (25 to 100** **mM); 5HT_2A_ receptor agonists: *α*-methyl-5HT (0.01 to 10 *μ*M), TCB-2 (0.001 to 10 *μ*M), and BRL54443 (0.01 to 10 *μ*M); 5HT_2B_ receptor agonists: norfenfluramine (0.01 to 10 *μ*M) and BW723C86 (0.01 to 1 *μ*M); and 5HT_1B_ agonist: CP93129 (0.001 to 10 *μ*M) were compared. Concentration response curves were randomized except responses to BW723C86 or TCB-2 that were always measured last since they were difficult to wash out. Responses to serotonin were measured in all studies whereas only two other serotonin receptor agonists were tested per sample. Effects of 5HT_2A_ and _2B_ receptor antagonists, MDL11939 (10** **nM) [20], and LY272015 (30** **nM) [12], respectively, on contractions to serotonin were obtained in aorta from ND and DB mice. To determine the contribution of rho kinase in contractions to serotonin, and 5HT_2A_ and _2B_ receptor agonists, responses to serotonin, *α*-methyl-5HT, TCB-2, norfenfluramine, and KCl were measured in the presence of H1152 (1 *μ*M), a specific rho kinase inhibitor [21, 22]. To test the role of endothelial-derived vasodilator substances in contractions to serotonin, in a subset of studies the endothelium was mechanically removed by gently rubbing the inner surface with suture, nitric oxide synthase (NOS) was inhibited with nitro-L-arginine (LNNA, 10 *μ*M), or cyclooxygenase was inhibited with indomethacin (10 *μ*M). Endothelial removal and NOS inhibition were confirmed by absence of relaxation to acetylcholine (0.1 to 10 *μ*M) following contraction to thromboxane mimetic U46619. In general, two aortic rings from each animal were used as controls and two were treated with a 5HT receptor antagonist, rho kinase inhibitor, LNNA, or indomethacin.

### 2.3. Western Blot Analysis

In separate animals, a single aorta (1 per sample) was isolated, flash frozen in liquid nitrogen, crushed, and mixed with a denaturing SDS buffer before sonication [23]. The homogenate was rotated for 2 hours before centrifugation (14,000 ×g, 10 minutes, 4°C). Protein concentration of the supernatant was determined by the bicinchoninic acid method. Equal amounts of protein were separated by SDS-PAGE gel electrophoresis. After blocking, immunoblotting was performed using anti-5HT_2A_R (1 : 500, Abcam, ab16028, Cambridge, MA), anti-5HT_2B_R (1 : 500, BD Biosciences, 556334), anti-5HT_1B_R (1 : 500, Abcam, ab13896), and anti-pan-actin (1 : 500, Sigma-Aldrich, A2066) followed by secondary antibodies conjugated with horseradish peroxidase. Immunoreactivity was visualized with enhanced chemiluminescence. Blots were digitized and normalized to actin for comparison (NIH Image).

### 2.4. Materials

All chemicals were purchased from Sigma-Aldrich except *α*-methyl-5HT, TCB-2 ((4-bromo-3,6-dimethoxybenzocyclobuten-1-yl)methylamine hydrobromide), BRL54443 (5-hydroxy-3-(1-methylpiperidin-4-yl)-1*H*-indole), BW723C86 (*α*-methyl-5-(2-thienylmethoxy)-1*H*-indole-3-ethanamine  hydrochloride), CP93129 (1,4-Dihydro-3-(1,2,3,6-tetrahydro-4-pyridinyl)-5*H*-pyrrol  [3,2-*b*]pyridin-5-one dihydrochloride), MDL119939 (*α*-Phenyl-1-(2-phenylethyl)-4-piperidinemethanol), and LY272015 (1-[(3,4-Dimethoxyphenyl) methyl]-2,3,4,9-tetrahydro-6-methyl-1*H*-pyrido  [3,4-*b*]indole hydrochloride), that were obtained from Tocris Bioscience, and H1152 ((S)-(+)-2-Methyl-1-[(4-methyl-5-isoquinolinyl)sulfonyl]  homopiperazine, 2HCl) and U46619 were obtained from Enzo Life Sci.

### 2.5. Statistical Analysis

Data are presented as mean ± SEM. Responses of multiple rings from a given animal treated similarly were averaged and “*n*” represents numbers of mice per group. Concentration response curves were compared by repeated measures or two way analysis of variance followed by Bonferroni post hoc test. EC_50_s, weights, blood glucose levels, and protein expression were compared by using an unpaired or paired *t*-test (Prism). Significance was defined as *P* < 0.05.

## 3. Results

### 3.1. Enhanced Serotonin-Induced Contractions in Arteries from Diabetics

**Figure 1:**
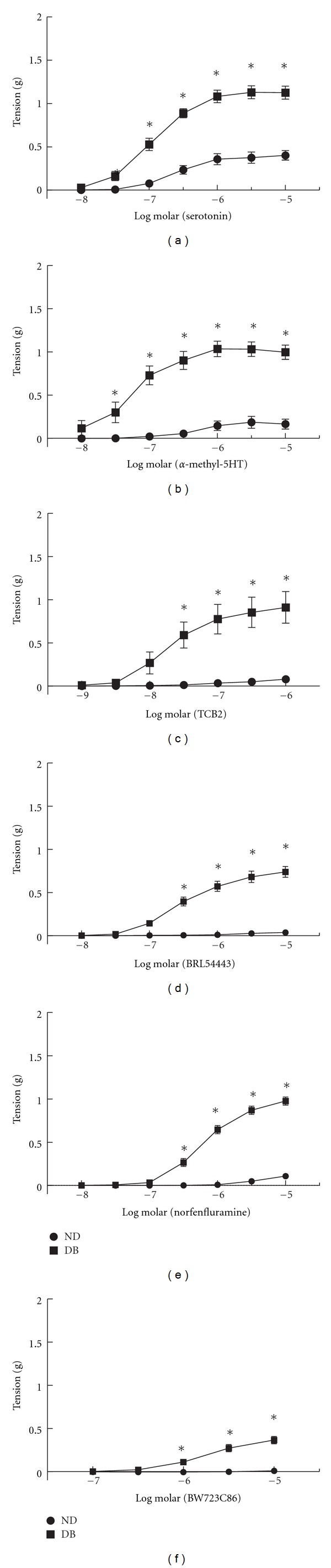
Contractions of aorta from non-diabetic (ND) and diabetic (DB) mice to serotonin (a); the 5HT_2A_ agonists *α*-methyl-5HT (b), TCB-2 (c), and BRL4443 (d); and the 5HT_2B_ agonists norfenfluramine (e) and BW723C86 (f). All data are mean ± SEM. *P < 0.05 versus ND.

Similar to our previous studies in non-diabetic mice [5, 16, 17], serotonin produced concentration-dependent contractions of arteries from ND (0.40 ± 0.05 g at 10 *μ*M, *n* = 17, Figure 1(a)). Contractions of arteries from DB mice were markedly augmented to serotonin (1.13 ± 0.08 g at 10 *μ*M, *n* = 14, Figure 1(a)). Although the maximal contractions were increased, the EC_50_'s for contractions to serotonin in arteries from DB compared to ND were not different (Table 2).

**Figure 2:**
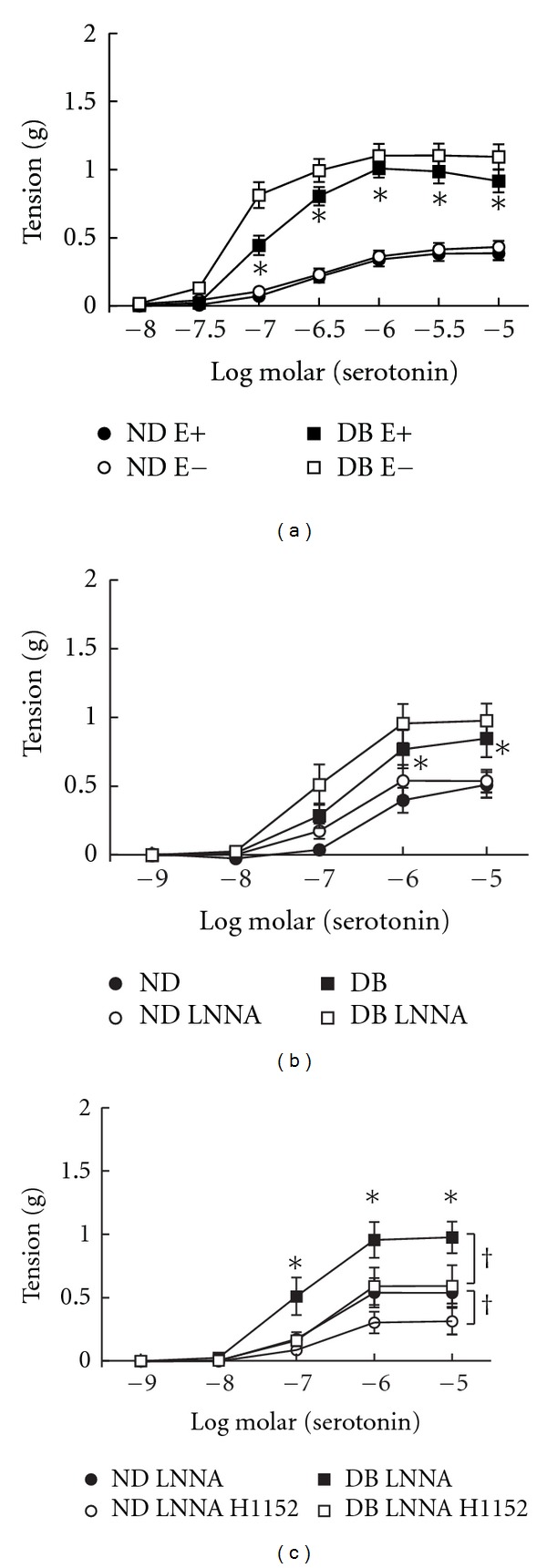
Relaxation of aorta from non-diabetics (ND) and diabetics (DB) to serotonin with (E+) and without (E−) endothelium (a), following inhibition of nitric oxide synthase with nitro-L-arginine (LNNA, b) and LNNA with H1152 (c). All data are mean ± SEM, *P < 0.05 versus ND, ^†^ *P* < 0.05 versus control.

To determine the role of endothelial-derived factors which can impact contractions to serotonin [5, 24], the endothelium was removed, NOS was inhibited with LNNA, or cyclooxygenase was inhibited with indomethacin. Relaxation in response to acetylcholine was modestly impaired in arteries from DB mice compared to ND (%relaxation at 10 *μ*M: ND 66 ± 4 versus DB 41 ± 7, *P* < 0.05) but the difference was abolished when endothelium was removed (%relaxation at 10 *μ*M: ND 7 ± 3 versus DB 7 ± 4) or NOS was inhibited with LNNA (%relaxation at 10 *μ*M: ND 28 ± 4 versus DB 37 ± 12). In contrast to previous studies in arteries from C57BL/6 mouse strain [5], neither endothelial removal (ND *n* = 22, DB *n* = 7) nor inhibition of NOS with LNNA (ND *n* = 7, DB *n* = 8) affected contractions to serotonin in ND or DB arteries (Figures 2(a) and 2(b)). Inhibition of cyclooxygenase with indomethacin also had no effect on contractions to serotonin in arteries from these ND and DB mice (data not shown). Thus the increased contractions to serotonin in these DB mice were not related to a change in substances derived from NOS or cyclooxygenase. Since the endothelium did not appear to impact contractions to serotonin in aorta of these mouse strains, all remaining studies were conducted in the presence of endothelium.

### 3.2. Contractions of Arteries to Selective Serotonin Receptor Agonists

To determine whether activation of specific serotonin receptors accounts for the difference in contractions in diabetics, we compared responses to several serotonin receptor agonists. We first compared responses to *α*-methyl-5HT (mixed 5HT_2A_, 5HT_2B_, and 5HT_2C_ agonist). Whereas responses of arteries from ND to *α*-methyl-5HT were modest, arteries from DB mice generated marked contractions to *α*-methyl-5HT (Figure 1(b)). The maximal contractions at 30 *μ*M *α*-methyl-5HT were fivefold greater in DB (1.03 ± 0.08 g, *n* = 12, *P* < 0.05 versus ND) compared to ND (0.19  ±  0.07 g, *n* = 11). Not only was the maximal contraction increased but the concentration response curve was shifted with a reduction in the EC_50_ (Table 2).

Whereas *α*-methyl-5HT produced modest contractions of ND arteries, ND arteries did not respond to either of the 5HT_2A_ receptor agonists, TCB-2 (*n* = 6, Figure 1(c)), or BRL54443 (*n* = 11, Figure 1(d)). Similar to *α*-methyl-5HT, contractions of DB arteries to TCB-2 (*n* = 6) and BRL54443 (*n* = 18) were markedly greater than ND (maximal contractions to TCB-2 0.91 ± 0.18 g* versus 0.08 ± 0.02 g; BRL54443 0.74 ± 0.06 g* versus 0.04 ± 0.01 g, *P < 0.05 ND versus DB). An EC_50_ for TCB-2 and BRL54443 could not be determined in arteries from ND mice. Thus, contractions in response to all 5HT_2A_ receptor agonists were modest in ND but robustly augmented in arteries from diabetics compared to non-diabetics.

To address the contribution of 5HT_2B_ receptor activation in response to serotonin, we compared responses of arteries from ND and DB mice to the  5HT_2B_ receptor agonists, norfenfluramine, and BW723C86. Neither norfenfluramine nor BW723C86 significantly contracted arteries from ND mice (Figures 1(e) and 1(f)). Contractions to both 5HT_2B_ receptor agonists were markedly enhanced in arteries from DB mice (Figures 1(e) and 1(f)). The maximal contraction to norfenfluramine at 10 *μ*M was 0.98 ± 0.05 g compared to 0.37 ± 0.04 g with BW732C86. The EC_50_ for norfenfluramine was shifted in arteries from DB mice compared to ND (Table 2). Since arteries from ND mice did not respond to BW723C86, an EC_50_ could not be determined. Even in arteries from DB mice, BW732C86 was less potent than other serotonin receptor agonists (Table 2). Thus, similar to 5HT_2A_ receptor agonists, responses to specific 5HT_2B_ receptor agonists were modest in non-diabetics but markedly increased in arteries from diabetics.

To determine whether the augmented contractions to serotonin may be due to a change in the contribution of 5HT_1B_ receptors, we compared responses to the 5HT_1B_ receptor agonist CP93129. CP93129 did not contract arteries from ND mice (*n* = 4, data not shown) and in contrast to both 5HT_2A_ and 5HT_2B_ agonists, arteries from DB mice did not contract to CP93129 either (*n* = 4, data not shown). Thus, both 5HT_2A_ and 5HT_2B_ receptors but not 5HT_1B_ receptors, in part, mediate contractions to 5HT in arteries from non-diabetics and were increased in arteries from diabetics.

### 3.3. Receptor Subtypes Mediating Contractions to Serotonin in Non-Diabetic and Diabetic Arteries

**Figure 3:**
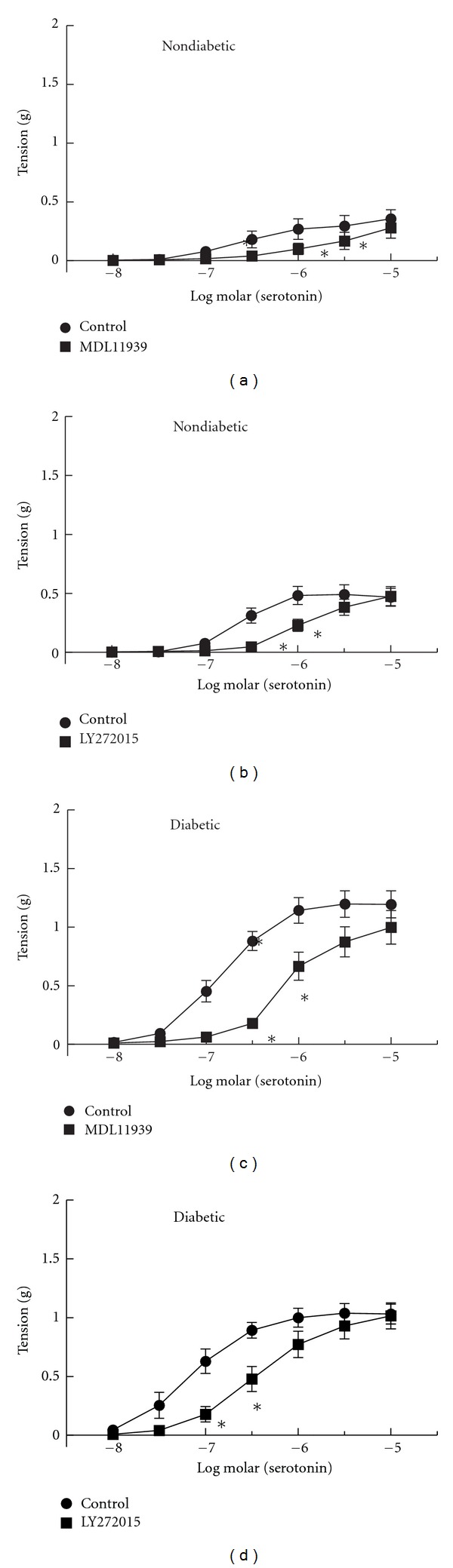
Contractions of aorta from non-diabetic (a and b) and diabetic (c and d) mice before (Control) and after inhibition of 5HT_2A_ receptors with MDL11939 (10 nM, (a) and (c)) or 5HT_2B_ receptors with LY272015 (30 nM, (b) and (d)). All data are mean ± SEM. *P<0.05 versus Control.

To determine the 5HT receptor subtypes mediating contractions to serotonin, we compared effects of specific receptor antagonists on responses of arteries from ND and DB mice. In arteries from ND mice, both the 5HT_2A_  receptor antagonist (MDL11939, 10 nM, *n* = 10) and 5HT_2B_ receptor antagonist (LY272015, 30 nM, *n* = 7) inhibited contractions to serotonin (Figures 3(a) and 3(b)). Both the 5HT_2A_ and 5HT_2B_ receptor antagonists had greater effects on serotonin-induced contractions in arteries from DB mice (Figure 3(c), *n* = 7 and Figure 3(d), *n* = 6). Thus, in arteries from both non-diabetic and diabetic leptin receptor deficient mice, activation of both 5HT_2A_  and 5HT_2B_ receptors mediates contractions to serotonin.

### 3.4. Expression of Serotonin Receptors in Non-Diabetic and Diabetic Arteries

**Figure 4:**
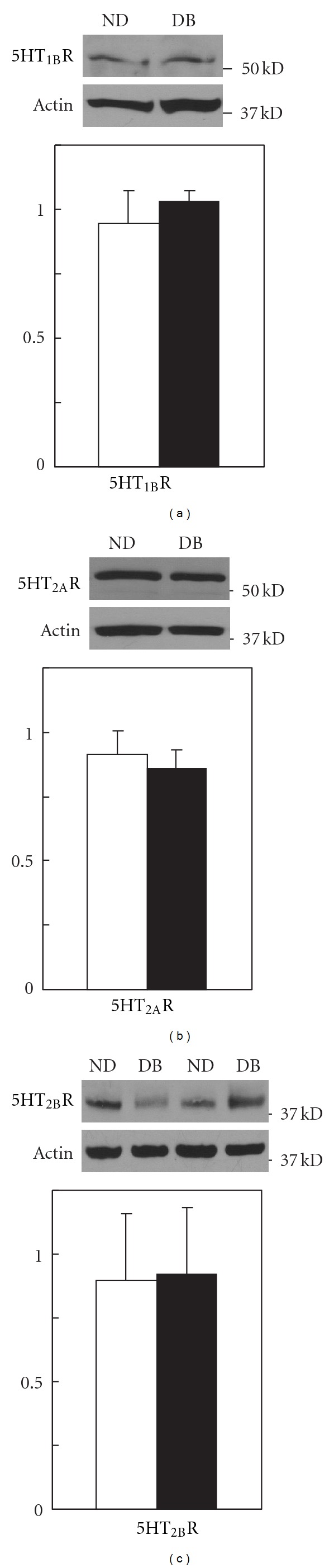
Representative western immunoblots of expression of 5HT_1B_ receptors (5HT_1B_R, (a)), 5HT_2A_R (b), 5HT_2B_R (c), and actin along with the average expression of each receptor normalized to actin in aorta from non-diabetics (open bars) and diabetics (closed bars). All data are mean ± SEM, *n* = 4 each.

To determine whether the augmented serotonin-mediated contractions in arteries from diabetics were due to changes in the expression of specific receptor subtypes, we compared expression of 5HT_2A_, 5HT_2B_, and 5HT_1B_ receptors in arteries from non-diabetic and diabetic mice (*n* = 4 each). There were no differences in the expression of either of the serotonin receptor subtypes that mediate contractions of arteries from non-diabetics and diabetics (5HT_2A_ and 5HT_2B_ receptors, Figures 4(b) and 4(c)). Unexpectedly, although 5HT_1B_ receptors do not appear to mediate vascular contractions to serotonin, 5HT_1B_ receptors were expressed in mouse arteries but were not different in ND and DB mice (Figure 4(a)). Thus, increased expression of specific serotonin receptor subtypes does not account for the augmented contractions to serotonin observed in arteries from diabetics.

### 3.5. Role of rho Kinase in Contractions to Serotonin Receptor Agonists

**Figure 5:**
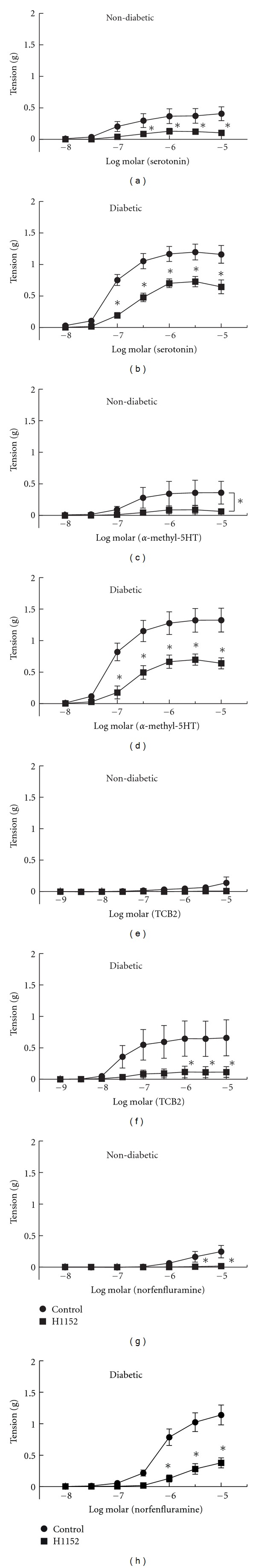
Contractions of aorta from non-diabetic (a c, e, and g) and diabetic (b, d, f, and h) mice to serotonin, *α*-methyl-5HT, TCB-2, and norfenfluramine before (Control) and after inhibition of rho kinase with H1152 (1 *μ*M). All data are mean ± SEM. *P < 0.05 versus Control.

We compared the contribution of rho kinase in the contractions to the specific 5HT_2A_ and 5HT_2B_ receptor agonists. Inhibition of rho kinase with H1152 reduced contractions of arteries from ND mice to serotonin (Figure 5(a), *n* = 11). Although contractions of arteries from ND mice to the 5HT_2A_ receptor agonist, *α*-methyl-5HT (*n* = 6), and the 5HT_2B_ receptor agonist, norfenfluramine (*n* = 8), were modest, both were inhibited by H1152 (1 *μ*M, Figures 5(c) and 5(g)). In DB mice, H1152 reduced contractions of arteries to serotonin (Figure 5(b), *n* = 8). Combined inhibition of NOS and rho kinase had a similar effect as H1152 alone on contractions to serotonin in arteries from either ND or DB mice (Figure 2(c)). Similar to ND mice, contractions of arteries from DB mice to *α*-methyl-5HT (Figure 5(d), *n* = 5), TCB-2 (Figure 5(f), *n* = 3), and norfenfluramine (Figure 5(h), *n* = 7) were reduced by inhibition of rho kinase with H1152 (Figure 5). Thus, the enhanced contractions to selective 5HT_2A_ and 5HT_2B_, receptor agonists were mediated, in part, through an increase in rho kinase activation.

### 3.6. Role of rho Kinase in Contractions in Response to KCl

**Figure 6:**
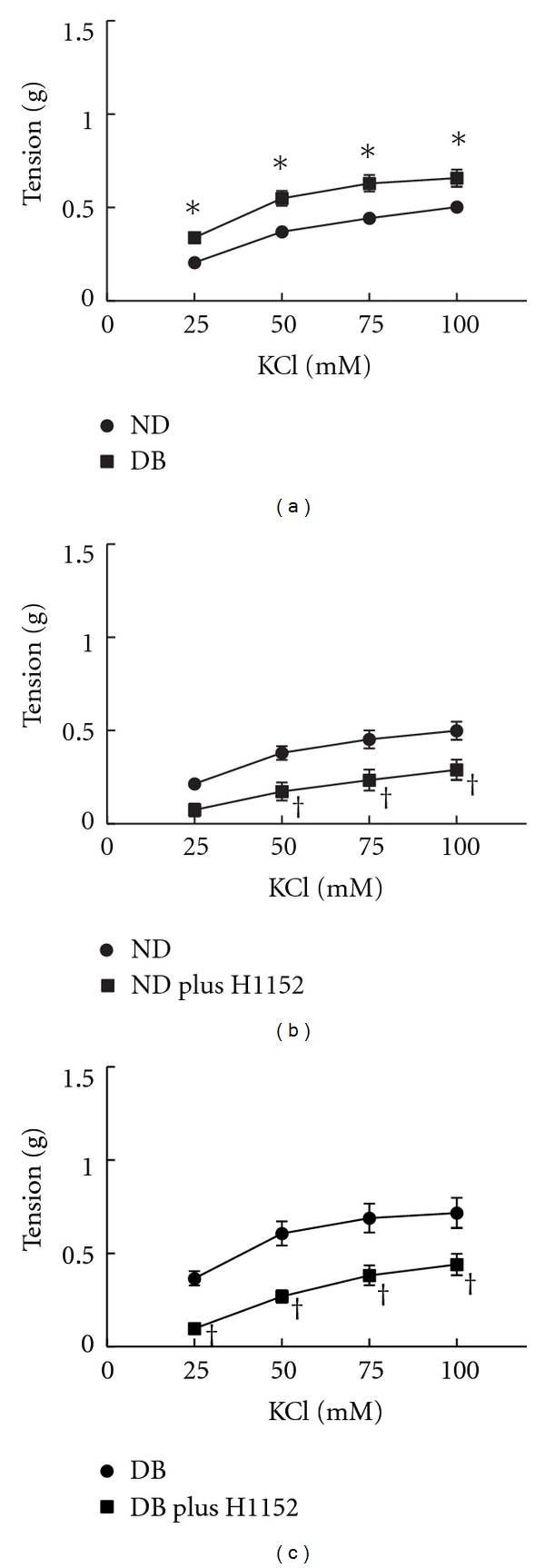
Contractions of aorta from non-diabetics (ND) and diabetic (DB) mice to KCl (a). Effect of inhibition of rho kinase with H1152 (1 *μ*M) on contractions to KCl in non-diabetics (b) or diabetics (c) compared to responses before H1152 (Control). All data are mean ± SEM. *P < 0.05 versus ND < 0.05 versus ND, ^†^ *P* < 0.05 versus Control.

In addition to serotonin, we compared contractions of arteries from ND and DB mice in response to receptor-independent membrane depolarization with KCl. KCl produced concentration dependent contractions of arteries from ND mice (Figure 6(a), *n* = 19). The magnitude of the contractions in arteries from ND was similar to responses to serotonin. Contractions to KCl were unaffected by the 5HT_2A_ and 5HT_2B_ receptor antagonists, MDL11939, and LY272015 (data not shown). Similar to serotonin, contractions to KCl were increased in arteries from DB mice (Figure 6(a), *n* = 20) though the magnitude of the shift was less than what was observed with serotonin (Figure 6(a)). As we and others have previously demonstrated in non-diabetic arteries [5, 25], contractions to KCl were reduced by inhibition of rho kinase with H1152 (1 *μ*M) in arteries from both ND (Figure 6(b), *n* = 6) and DB mice (Figure 6(c), *n* = 8). Thus, contractions in response to both serotonin receptor activation and receptor-independent membrane depolarization were mediated, in part, by rho kinase and were increased in arteries from DB mice.

## 4. Discussion

The present study demonstrates that the augmented contractions of arteries in a model of type 2 diabetes are primarily due to changes in signaling pathways within smooth muscle that regulate calcium sensitivity of contractile proteins rather than a change in endothelial-derived vasoactive factors or specific serotonin receptor subtypes. In both non-diabetic and diabetic mice, contractions to serotonin were mediated by activation of both 5HT_2A_ and 5HT_2B_ receptors. Despite detectable expression levels, 5HT_1B_ receptors were not involved in the contraction to serotonin in either ND or DB since there was no response to the 5HT_1B_ receptor agonist. The function of 5HT_1B_ receptors in these blood vessels is not known. Abnormal endothelial factors derived from NOS and cyclooxygenase did not contribute to the augmented contractions to serotonin since endothelial removal; LNNA and indomethacin did not significantly affect contractions to serotonin of aorta from either non-diabetic or diabetic mice. Contractions to all 5HT_2A_ and 5HT_2B_ receptor agonists were increased and inhibition of both 5HT_2A_ and 5HT_2B_ receptors shifted contractions to serotonin in arteries from DB mice. This occurred in the absence of a change in the expression of either receptor subtype in DB arteries. Contractions to serotonin and both 5HT_2A_ and 5HT_2B_ receptor agonists were reduced following blockade of rho kinase. Receptor-independent contractions of DB arteries to KCl were also augmented and mediated, in part, by rho kinase. These data support the conclusion that a change in rho kinase regulation of calcium sensitivity and not 5HT receptor expression likely mediates the hypercontractility of arteries from diabetics.

Our data suggest that in addition to 5HT_2A_ receptors, 5HT_2B_ receptors also play a significant role. Although selectivity of agonists and antagonists is dependent on concentration, it should be noted that both *α*-methyl-5HT and BRL54443 have affinity for other 5HT receptor subtypes as well as 5HT_2A_ receptors [26, 27]. For this reason, we also compared responses to a more specific  5HT_2A_  receptor agonist, TCB-2 [28]. Despite differences in receptor specificity, all arteries from diabetic mice generated similar contractions to the 5HT_2A_ receptor agonists. In contrast, despite similar binding affinity for the 5HT_2B_ receptor in cultured cells [29, 30], contractions to 5HT_2B_ receptor agonist norfenfluramine were much greater than contractions to BW723C86, but were similar to those reported by Watts' and coworkers [4, 31]. Both norfenfluramine and BW723C86 have affinity for 5HT_2A_ and 5HT_2C_ receptors [26]. Despite similar binding affinities, differences in the magnitude of contractions to norfenfluramine and BW723C86 highlight issues with extrapolation of binding affinities derived from cell systems to intact arteries. Direct comparisons may not be valid since binding affinity alone does not determine the overall potency of an agonist in intact tissues. Because of potential non-specific effects, we utilized low concentrations and multiple, structurally distinct receptor agonists and antagonists. Given these limitations, our studies still provide strong evidence for a role for both 5HT_2A_ and 5HT_2B_ receptors and not 5HT_1B_ receptors in contractions to serotonin in arteries from both non-diabetic and diabetic mice. Development of more selective agonists and antagonists or mice with targeted gene deletion of specific serotonin receptor subtypes will provide valuable tools to further determine the receptor subtypes mediating hypercontractions of arteries in diabetics.

Vascular responses to serotonin vary with vessel size, tissue source of arteries, and receptor subtype. Although in the present study, mouse aorta did not respond to a specific 5HT_1B_ receptor agonist, serotonin-induced contractions of human internal thoracic [32], coronary [33], and temporal arteries [34] are mediated, in part, by activation of 5HT_1B_ receptors. Variants in the gene encoding the 5HT_1B_ receptor contributed to the magnitude of the contraction to 5HT in human arteries [34]. Genetic variability may also contribute to responses in mice as evident in the present and previous studies of arteries from non-diabetic mice. Though originally derived from the Black6 strain, the non-diabetic mouse used in these studies was the C57BLKS/J, the background strain in which the BKS.Cg-*m*+/+*Lepr* ^*db*^/J was derived. Contractions to 5HT were greater in non-diabetic C57BL/6J compared to C57BLKS/J mice, were mediated by activation of the 5HT_2A_ receptor, and were modulated by nitric oxide released from eNOS within endothelium [4, 5, 17]. The difference in the magnitude of the contractions, contribution of specific receptor subtypes, and role of endothelium in response to serotonin in non-diabetic C57BLKS/J mice in the present study is likely related to differences in mouse strain. Differences in the magnitude of contractions to 5HT appear specific since contractions to KCl were similar in both the C57BL/6J and C57BLKS/J strains [5]. Strain differences in vascular responses are not unprecedented. Arterial blood pressure, vascular responses to acetylcholine, development of atherosclerosis in response to high fat diet and other phenotypes vary significantly in different mouse strains [35, 36]. Although many investigators use heterozygous leptin receptor deficient *Dock7* ^*m*^+/+*Lepr* ^*db*^ or homozygous *Dock7* ^*m*^+/*Dock7* ^*m*^+ littermates as controls for the leptin receptor deficient mouse model, both of these mice have subtle phenotypes that might impact results. 

The model of diabetes used in the present study was dependent upon abnormal leptin signaling which might contribute to vascular dysfunction. While interactions between leptin and serotonin signaling impact appetite and neural function, there are no studies describing an interaction between leptin and serotonin in the regulation of vascular function. It is unlikely that the findings of the present study are a direct result of leptin receptor deficiency since abnormal vascular responses to serotonin are observed in other leptin-independent murine models of type 1 and type 2 diabetes [16, 19, 37]. Although leptin signaling may contribute to blood pressure regulation which can impact vascular reactivity, there is little consensus regarding blood pressure in leptin receptor deficient mice with reports of reduced [38], no change [39, 40], and elevated blood pressures [38, 39, 41–43] compared to wild type mice. In studies where systemic blood pressure was significantly elevated in leptin receptor deficient mice, the magnitude was generally modest (~10 mmHg) [41–43]. Thus, although the abnormal vascular responses in arteries from db/db mice may, in part, be due to an elevation in blood pressure, an elevated blood pressure is unlikely to fully explain the abnormalities.

An alternative mechanism for the vascular dysfunction involves insulin-induced effects on receptor localization at the plasma membrane. Increased levels of insulin induce internalization of 5HT_2A_ receptors in smooth muscle of saphenous veins [44]. Insulin levels in db/db mice are dependent upon age with increases peaking 5–10 times higher than non-diabetic mice at 4-5 weeks of age and subsequently declining til they are normal at 25 weeks of age [45–47]. Although insulin-induced effects on receptor localization may occur in this model, it appears to be unlikely since this mechanism would reduce contractions in younger db/db mice when insulin levels are highest and restore contractions to near normal at 25 weeks of age when levels are near normal, opposite to what we observed. Insulin may affect localization of other signaling components at the plasma membrane. We propose that chronic hyperglycemia most likely plays a major role in the altered serotonin signaling in arteries observed in this study.

Although serotonin was used in this study as a tool to examine mechanisms for large artery dysfunction in diabetes, the clinical relevance of these studies should be noted. The majority of serotonin is found in platelets under normal conditions, but circulating levels as high as 1 *μ*M have been measured in the coronary sinus following thrombus formation [48, 49]. Vasospasm of large arteries associated with thrombus formation and postangioplasty can be inhibited by serotonin receptor blockers [50]. Circulating levels of serotonin are increased similarly (700 nM) in diabetics [48] and are well within the range used in our studies. Thus, abnormal serotonin levels may contribute to the larger artery dysfunction and increased incidence of vascular complications in diabetics.

## Figures and Tables

**Table 1:** Characteristics of non-diabetic and diabetic mice.

	Weight (g)	Glucose (mg/dL)	Age (weeks)	*n*
Nondiabetic	27.5 ± 0.4	161 ± 8	26 ± 3	42
Diabetic	50.1 ± 0.8*	508 ± 13*	20 ± 1	47

Mean ± SEM, *P < 0.05 versus non-diabetic.

**Table 2:** Negative log EC_50_ values [M] for serotonin receptor agonists in arteries from non-diabetic and diabetic mice.

Agonist	Non-diabetic	*n*	Diabetic	n
5HT	6.61 ± 0.14	17	6.96 ± 0.09	14
*α*-Methyl-5HT	6.33 ± 0.24	11	7.21 ± 0.17*	12
TCB-2	ND	6	7.78 ± 0.28	6
BRL54443	ND	11	6.54 ± 0.11	18
Norfenfluramine	5.39 ± 0.29	21	6.20 ± 0.05*	29
BW723C86	ND	6	5.73 ± 0.13	12

Mean ± SEM, ND: not determined, *P < 0.05 versus non-diabetic.
